# A study on the current status and associated factors of artificial intelligence literacy among undergraduate nursing students

**DOI:** 10.3389/fpubh.2026.1930077

**Published:** 2026-09-22

**Authors:** Fangyun Li, Kexin Feng, Shuangshuang Wang, Songmei Xue, Jinhua Zhang, Wei Li

**Affiliations:** College of Nursing, North Henan Medical University, Henan, China

**Keywords:** artificial intelligence literacy, creative mindset, critical thinking disposition, nursing education, nursing students

## Abstract

**Introduction:**

The primary objective of this study was to investigate undergraduate nursing students’ present level of artificial intelligence (AI) literacy and related issues, as well as to offer resources that nursing educators may use to create focused intervention methods.

**Methods:**

From October to December 2025, 389 third-year undergraduate nursing students from three Henan Province universities were enlisted as survey respondents using a convenience sampling technique. A general information questionnaire, the Chinese College Students’ AI Literacy Scale, the Critical Thinking Disposition Inventory, and the Creative Mindset Questionnaire were used to gather data. Pearson correlation analysis, theory-driven multiple linear regression analysis, and exploratory indirect-association analysis using PROCESS v4.1 Model 4 were conducted.

**Results:**

Undergraduate nursing students’ overall AI literacy score was 55.71 ± 7.34. AI literacy showed a positive correlation with both creative mindset (*r* = 0.341, *p* < 0.01) and critical thinking disposition (*r* = 0.544, *p* < 0.01). Regression analysis revealed that attitude toward AI, prior AI-related education, critical thinking disposition, and creative growth mindset were statistically significant predictors of AI literacy (*p* < 0.05). A statistically significant indirect relationship between critical thinking disposition and AI literacy through creative growth mindset was found by Bootstrap analysis, accounting for roughly 21.51% of the overall link.

**Conclusion:**

The average AI literacy score of undergraduate nursing students was 55.71 ± 7.34, with variance across AI literacy characteristics. The important associations of AI literacy with critical thinking disposition and creative growth mindset should be acknowledged in nursing education. AI education should not be limited to knowledge imparting and skill training but should integrate the cultivation of critical evaluation and creative growth beliefs to enhance nursing students’ AI literacy.

## 1 Introduction

Generative Artificial Intelligence (AI) has rapidly entered higher education learning contexts and continues to transform the ways students access information and complete academic tasks (1). However, while generative AI enhances learning efficiency, it also raises concerns such as indiscriminate use and privacy risks (2). Therefore, for nursing students, the advent of generative AI demands not only the ability to use such tools but also the capacity to understand, evaluate, and responsibly apply AI throughout their professional learning process. Artificial intelligence literacy is a key concept for measuring whether individuals can effectively engage in this process. AI literacy encompasses not only an understanding of basic AI concepts and tool usage but also the ability to evaluate AI-generated content and its limitations, as well as the capacity for ethical judgment and risk prevention during application (3).

Current research on AI literacy among university and medical students has primarily focused on describing literacy levels and examining factors such as interest, attitudes, technology-use experience, and educational background (4, 5). Studies in nursing have similarly emphasized the assessment of AI literacy, the development of educational strategies, and preliminary identification of associated factors (6, 7). However, the theoretical mechanisms through which educational experience, cognitive dispositions, and learning beliefs may jointly relate to AI literacy remain insufficiently explained. To address this gap, the present study draws on Social Cognitive Theory, which proposes that competency development emerges through reciprocal relationships among environmental conditions, personal cognitive and motivational factors, and individuals’ engagement with learning activities (8). Within this framework, prior AI-related education represents an environmental learning exposure because it may provide structured knowledge, guided practice, feedback, and normative guidance (9). Attitude toward AI represents a motivational orientation that may influence students’ willingness to engage with AI-related learning. Critical thinking disposition represents a cognitive tendency to question information, evaluate evidence, identify inconsistencies, and assess the credibility and limitations of AI-generated content (2, 10). Creative mindset comprises beliefs about whether creativity is malleable and can be developed through learning and practice (creative growth mindset) or is relatively stable (creative fixed mindset) (11–13). AI literacy is therefore conceptualized as a multidimensional competency involving awareness, practical use, critical evaluation, and ethical application of AI (3, 14).

Creative growth mindset was specifically examined as a potential indirect pathway between critical thinking disposition and AI literacy. This creativity-specific belief was considered relevant because effective engagement with generative AI may involve iterative problem solving, evaluation and revision of generated outputs, exploration of alternative solutions, and adaptation of strategies. Students who view creativity as developable may therefore be more willing to persist, experiment, seek feedback, and revise their approaches when working with unfamiliar AI-supported tasks. Critical thinking may enable nursing students to recognize inaccuracies, unsupported conclusions, algorithmic limitations, biases, and ethical risks in AI-generated information. Nevertheless, identifying such problems may not automatically result in sustained learning or more competent AI use. Students who believe that their creative abilities can be improved may be more likely to interpret errors and difficulties as opportunities for development, persist when learning unfamiliar technologies, seek feedback, revise ineffective strategies, and engage in repeated reflective practice (15–17). In this way, a creative growth mindset may help translate critical evaluation into continued learning and adaptive engagement with AI, thereby providing a theoretical explanation for its potential indirect association with AI literacy. By contrast, a creative fixed mindset represents the belief that creative abilities are relatively stable and was examined as a contrasting belief orientation rather than as an assumed adaptive pathway. Accordingly, the proposed framework considers AI education experience as an environmental correlate, attitudes and mindset as personal motivational factors, critical thinking disposition as a cognitive factor, and AI literacy as the multidimensional competency outcome. Based on this framework, the study examined whether creative growth mindset statistically accounted for part of the association between critical thinking disposition and perceived AI literacy.

Therefore, this study targets undergraduate nursing students to investigate their current AI literacy status and to analyze the relationships among attitudes, AI education experience, critical thinking disposition, creative mindset, and AI literacy. Furthermore, it examines whether creative growth mindset statistically accounts for part of the association between critical thinking disposition and AI literacy. The findings aim to provide references for nursing educators to design AI education that integrates technical application, critical thinking, learning beliefs, and ethical judgment within the context of generative AI.

## 2 Subjects and methods

### 2.1 Subjects

Undergraduate nursing students from three universities in Henan Province were chosen as survey respondents between October and December 2025 using a convenience sampling technique. The requirements for inclusion were: (1) voluntary participation and informed consent; and (2) third-year nursing majors. Students who missed class throughout the study session due to leave were not included. For study planning, the sample size was estimated conservatively based on eight prespecified candidate independent variables. Using the criterion of at least 15 participants per candidate variable and allowing for a 20% increase for potentially invalid or incomplete questionnaires, the minimum target sample size was 144 participants. The final theory-driven multiple regression model included six predictors. A total of 389 valid questionnaires were ultimately included in the analysis, exceeding the minimum target sample size. In all, 389 valid questionnaires were gathered for this investigation. The North Henan Medical University Ethics Committee gave its approval to this study (NHMU202510-01).

### 2.2 Survey instruments

#### 2.2.1 General information questionnaire

Nursing students’ gender, interest in AI, attitudes toward AI, frequency of use of AI technology in the previous year, and whether they had received AI-related instruction were all covered in a self-designed questionnaire.

#### 2.2.2 Artificial intelligence literacy scale for Chinese college students (AILS-CCS)

Conceived by Ma et al. (18) in 2024, the AILS-CCS aims to assess AI literacy in Chinese university students. It encompasses four facets: Awareness, Usage, Evaluation, and Ethics. Awareness evaluates students’ comprehension of fundamental AI ideas and principles; Utilisation assesses their capacity to implement AI instruments in education and daily challenges; Assessment gauges their capacity to critically evaluate AI-generated material and identify the constraints of AI instruments; while Ethics examines the appropriate utilisation of AI, encompassing compliance with ethical standards and cognisance of security threats. The instrument comprises 15 items evaluated on a 5-point Likert scale (1 = strongly disagree, 5 = strongly agree), yielding total scores between 15 and 75. Elevated scores indicate enhanced AI literacy. Construct validity was assessed using confirmatory factor analysis, which demonstrated an acceptable model fit (CMIN/df = 2.358, RMSEA = 0.076, CFI = 0.941, NFI = 0.903). Composite reliability (CR) values ranged from 0.849 to 0.890, and average variance extracted (AVE) values ranged from 0.587 to 0.676, exceeding the commonly recommended thresholds of C*R* > 0.70 and AVE ≥ 0.50 (19). In the current investigation, the overall Cronbach’s α was 0.91, indicating outstanding internal consistency.

#### 2.2.3 Critical thinking disposition scale (CTDS)

Sosu et al. (14) created the Critical Thinking Disposition Scale in 2013 to evaluate individuals’ inclination to utilise critical thinking in problem-solving and educational contexts. The instrument comprises 11 items evaluated on a 5-point Likert scale. Total scores vary between 11 and 55, with elevated scores indicating a more robust critical thinking temperament. Scores ranging from 11 to 34 signify a low temperament, scores from 35 to 44 denote a moderate disposition, and scores between 45 and 55 reflect a high disposition. The initial scale exhibited strong reliability, evidenced by a Cronbach’s α coefficient of 0.82. In the current research, the Cronbach’s α coefficient was measured at 0.84, signifying strong internal consistency.

#### 2.2.4 Creative mindset scale (CMS)

The Creative Mindset Scale (CMS) (12) developed by Karwowski was translated into Chinese by Zhou et al. (20) and was adopted to measure individuals’ beliefs about creativity. There are 10 items in the scale, split into two subscales: Creative Fixed Mindset (5 items) and Creative Growth Mindset (5 items). Items are rated on a 7-point Likert scale and concern how much people believe that creativity is either a fixed ability (e.g., “Creativity is something you are born with - it can’t be learned”) or an ability that can be developed by effort and practice (e.g., “Creativity can be learned through effort and practice”). Cronbach’s α values for the Creative Fixed Mindset subscale and Creative Growth Mindset subscale were 0.84 and 0.70, respectively. The Cronbach’s α values for the overall scale, Creative Growth Mindset subscale and Creative Fixed Mindset subscale were found to be 0.86, 0.81 and 0.83, respectively, in the present study.

### 2.3 Data collection methods

The research team collected data using an online questionnaire format by distributing a Sojump QR code to the study participants. The questionnaire provided a detailed description of the research purpose and privacy protection measures and emphasized the voluntary nature of participation. Participants completed the questionnaire independently after giving full informed consent. The entire process was conducted anonymously, and the data were used solely for academic research. The Sojump system automatically validated logical skip patterns and required items to ensure data completeness. Upon collecting, questionnaires deemed invalid due to excessively brief response durations or repetitive consecutive answers were eliminated. A total of 421 questionnaires were received, with 389 being legitimate, resulting in a final effective response rate of 92.4%.

### 2.4 Statistical methods

Statistical characterisation and examination were conducted via SPSS 27.0 software. The Shapiro–Wilk test validated data normality, while Levene’s test evaluated the homogeneity of variance. Measurement data exhibiting a normal distribution were expressed as mean ± standard deviation (x̄ ± s). Variations among groups were evaluated by independent-samples t-tests and one-way analysis of variance (ANOVA), when suitable. Pearson correlation analysis was conducted to assess the associations between continuous variables. Multiple linear regression analysis was performed to examine factors independently associated with AI literacy, with the specified predictors entered simultaneously. Predictors were specified *a priori* based on the conceptual framework and their theoretical relevance to AI literacy rather than selected according to statistical significance in the univariate analyses. Accordingly, interest in AI, attitude toward AI, receipt of AI-related education, critical thinking disposition, creative growth mindset, and creative fixed mindset were entered simultaneously into the multiple linear regression model. Preliminary univariate and correlation analyses were used to describe bivariate relationships rather than as an automatic statistical variable-selection procedure. The evaluation of regression assumptions involved standardised residual-versus-predicted-value plots to examine linearity and homoscedasticity, a histogram and normal P–P plot to assess residual normality, the Durbin–Watson statistic to determine independence of residuals, and variance inflation factors (VIFs) to investigate multicollinearity. A predetermined parallel indirect-association analysis was conducted utilising PROCESS v4.1 Model 4, incorporating both creative growth mindset and creative fixed mindset concurrently as parallel mediating factors. Both variables were preserved in the model regardless of the statistical significance of their respective paths, and bootstrap confidence intervals were employed to assess the particular indirect correlations, with the significance threshold established at α = 0.05. Due to the measurement of all variables at a singular time point, the inferred indirect relationships were regarded as cross-sectional statistical trends rather than indicators of temporal or causal mediation. An exploratory factor analysis without rotation was conducted, and the variation accounted for by the initial factor was analysed. A percentage lower than 40% was deemed suggestive of the absence of substantial common method bias.

## 3 Results

### 3.1 Scores of AI literacy, critical thinking disposition, and creative mindset among undergraduate nursing students

Table 1 presents the descriptive statistics about AI literacy, critical thinking disposition, and creative mindset among undergraduate nursing students. The average score of AI literacy was 55.71 ± 7.34, and the average score of items was 3.71 ± 0.49. Among the AI literacy dimensions, Ethics had the highest mean score for items (3.99 ± 0.57), and Awareness had the lowest (3.43 ± 0.71). The mean score of critical thinking disposition was 42.90 ± 5.98, and creative mindset was 45.19 ± 9.07. Harman’s unidimensional assessment revealed multiple factors had eigenvalues exceeding one. The initial unrotated factor accounted for 31.8% of the overall variation, falling short of the commonly accepted 40% threshold, suggesting that common method bias was improbable in significantly influencing the study outcomes.

**Table 1:** Scores of AI literacy, critical thinking disposition, and creative mindset among undergraduate nursing students (*n* = 389).

Item	Total score (x̄ ± s)	Mean item score (x̄ ± s)
AI literacy	55.71 ± 7.34	3.71 ± 0.49
Awareness	13.72 ± 2.84	3.43 ± 0.71
Usage	11.19 ± 1.80	3.73 ± 0.60
Evaluation	14.84 ± 2.33	3.71 ± 0.58
Ethics	15.96 ± 2.29	3.99 ± 0.57
Critical thinking disposition	42.90 ± 5.98	3.90 ± 0.54
Creative mindset	45.19 ± 9.07	4.52 ± 0.91
Creative fixed mindset	20.22 ± 6.58	4.04 ± 1.32
Creative growth mindset	24.97 ± 4.71	4.99 ± 0.94

### 3.2 Univariate analysis of AI literacy among undergraduate nursing students

There were significant differences in AI literacy scores by interest in AI (*p* < 0.001), attitude toward AI (*p* < 0.001) and previous AI-related schooling (*p* < 0.001). A good attitude toward AI and receiving AI-related education were substantially associated with greater AI literacy scores among participants. In contrast, no significant differences were found related to gender (*p* = 0.996); however, AI literacy differed significantly across frequencies of AI technology use during the previous year (*F* = 4.019, *p* = 0.003) (Table 2).

**Table 2:** Univariate analysis of AI literacy among undergraduate nursing students.

Item	*n* (%)	AI literacy (x̄ ± s)	F/t	*P*
Gender			−0.005	0.996
Male	64 (16.5%)	55.70 ± 8.63		
Female	325 (83.5%)	55.71 ± 7.07		
Interest in AI			−4.097	<0.001
Yes	353 (90.7%)	56.18 ± 7.03		
No	36 (9.3%)	52.03 ± 8.65		
Attitude toward AI			9.266	<0.001
Positive	237 (60.9%)	56.73 ± 7.28		
Neutral	150 (38.6%)	54.28 ± 6.94		
Negative	2 (0.5%)	42.50 ± 16.26		
Frequency of AI technology use in the past year.			4.019	0.003
Daily	88 (22.6%)	55.91 ± 7.08		
Weekly	189 (48.6%)	56.50 ± 6.95		
Monthly	54 (13.9%)	55.81 ± 7.19		
Occasionally	51 (13.1%)	53.41 ± 7.01		
Almost never	7 (1.8%)	47.71 ± 15.40		
Received AI-related education			−6.356	<0.001
Yes	232 (59.6%)	57.56 ± 6.67		
No	157 (40.4%)	52.97 ± 7.44		

### 3.3 Correlation analysis of AI literacy, critical thinking disposition, and creative mindset among undergraduate nursing students

AI literacy was moderately and positively correlated with critical thinking disposition (*r* = 0.544, *p* < 0.001), creative mindset (*r* = 0.341, *p* < 0.001), and creative growth mindset (*r* = 0.449, *p* < 0.001). Critical thinking disposition was also positively associated with both creative mindset (*r* = 0.404, *p* < 0.001) and creative growth mindset (*r* = 0.601, *p* < 0.001). Creative Fixed mindset showed only weak positive correlations with AI literacy and critical thinking disposition (Table 3).

**Table 3:** Correlations (r) among AI literacy, critical thinking disposition, and creative mindset among undergraduate nursing students.

Item	1	2	3	4	5
1. AI literacy	1.00				
2. Critical thinking disposition	0.544**	1.00			
3. Creative mindset	0.341**	0.404**	1.00		
4. Creative growth mindset	0.449**	0.601**	0.716**	1.00	
5. Creative fixed mindset	0.149**	0.121*	0.867**	0.273**	1.00

**p < 0.01; *p < 0.05.

### 3.4 Multiple linear regression analysis of factors associated with AI literacy among undergraduate nursing students

A concurrent multiple linear regression analysis was conducted to investigate the independent relationship between interest in AI, attitude toward AI, previous AI-related education, critical thinking disposition, creative growth mindset, and creative fixed mindset with AI literacy, using the overall AI literacy score as the dependent variable. For dichotomous variables (interest in AI and receipt of AI-related education), assignment was made as “No = 0, Yes = 1”. For ordinal categorical variables (attitude toward AI), values were assigned in ascending order of rank. Scores for critical thinking disposition, creative growth mindset, and creative fixed mindset were entered as raw values, as shown in Table 4.

**Table 4:** Multiple linear regression analysis of factors associated with AI literacy among undergraduate nursing students (*n* = 389).

Independent variable	B	SE	Standardized β	*t*	*p*	95% CI for B	VIF
Constant	22.975	2.385	—	9.633	<0.001	18.286 to 27.664	—
Interest in AI (Yes vs. No)	2.034	2.101	0.041	0.968	0.334	−2.097 to 6.165	2.167
Attitude toward AI	2.396	0.626	0.095	3.827	<0.001	1.165 to 3.627	2.12
Received AI-related education (Yes vs. No)	3.047	0.618	0.204	4.93	<0.001	1.832 to 4.262	2.054
Critical thinking disposition	0.48	0.063	0.391	7.619	<0.001	0.356 to 0.604	2.63
Creative Growth mindset	0.245	0.083	0.157	2.952	0.003	0.082 to 0.408	2.727
Creative Fixed mindset	0.05	0.047	0.045	1.064	0.288	−0.042 to 0.142	2.086

B = unstandardized regression coefficient; SE = standard error; β = standardized regression coefficient; CI = confidence interval; VIF = variance inflation factor. Coding: interest in AI and AI-related education, No = 0 and Yes = 1. Attitude toward AI was entered as an ordinal variable. Model summary: R^2^ = 0.379, adjusted R^2^ = 0.369; F (6, 382) = 38.879, *p* < 0.001.

Regression diagnostics suggested that no serious breaches of model assumptions occurred. The residuals were fairly normally distributed as evidenced by the histogram and normal P–P plot, and the standardised residual against projected value plot did not exhibit any evident systematic pattern. The Durbin-Watson score was 1.94, suggesting no serious autocorrelation and supporting independence of residuals. The VIF values ranged from 2.054 to 2.727, indicating no significant multicollinearity.

### 3.5 Parallel indirect-association analysis of critical thinking disposition and creative mindset on AI literacy among undergraduate nursing students

Based on Social Cognitive Theory, this study evaluated whether the creative growth mindset and creative fixed mindset significantly mediated the cross-sectional relationship between critical thinking disposition and AI literacy. We tested a prespecified cross-sectional indirect association model, adjusted for theoretically important factors like gender, attitude toward AI, and receipt of AI-related education. The specific indirect relationship via creative fixed mindset was not significant; however, creative fixed mindset was kept in the prespecified parallel model regardless of significance. The resulting parallel indirect-association model incorporating both creative growth mindset and creative fixed mindset is shown in Figure 1. The bootstrap approach was applied to estimate the specific indirect associations through creative growth mindset and creative fixed mindset, and the findings were shown in Table 5. The indirect relationship through creative growth mindset had statistical support; however, the particular indirect association through creative fixed mindset did not. The direct relationship contributed 78.49% of the total association, whereas the entire indirect association through both creative mindset contributed 21.51%; the particular indirect associations through creative growth mindset and creative fixed mindset contributed 20.69 and 0.82%, respectively. The data show a statistically significant indirect pattern but do not show temporal or causal mediation.

**Figure 1:**
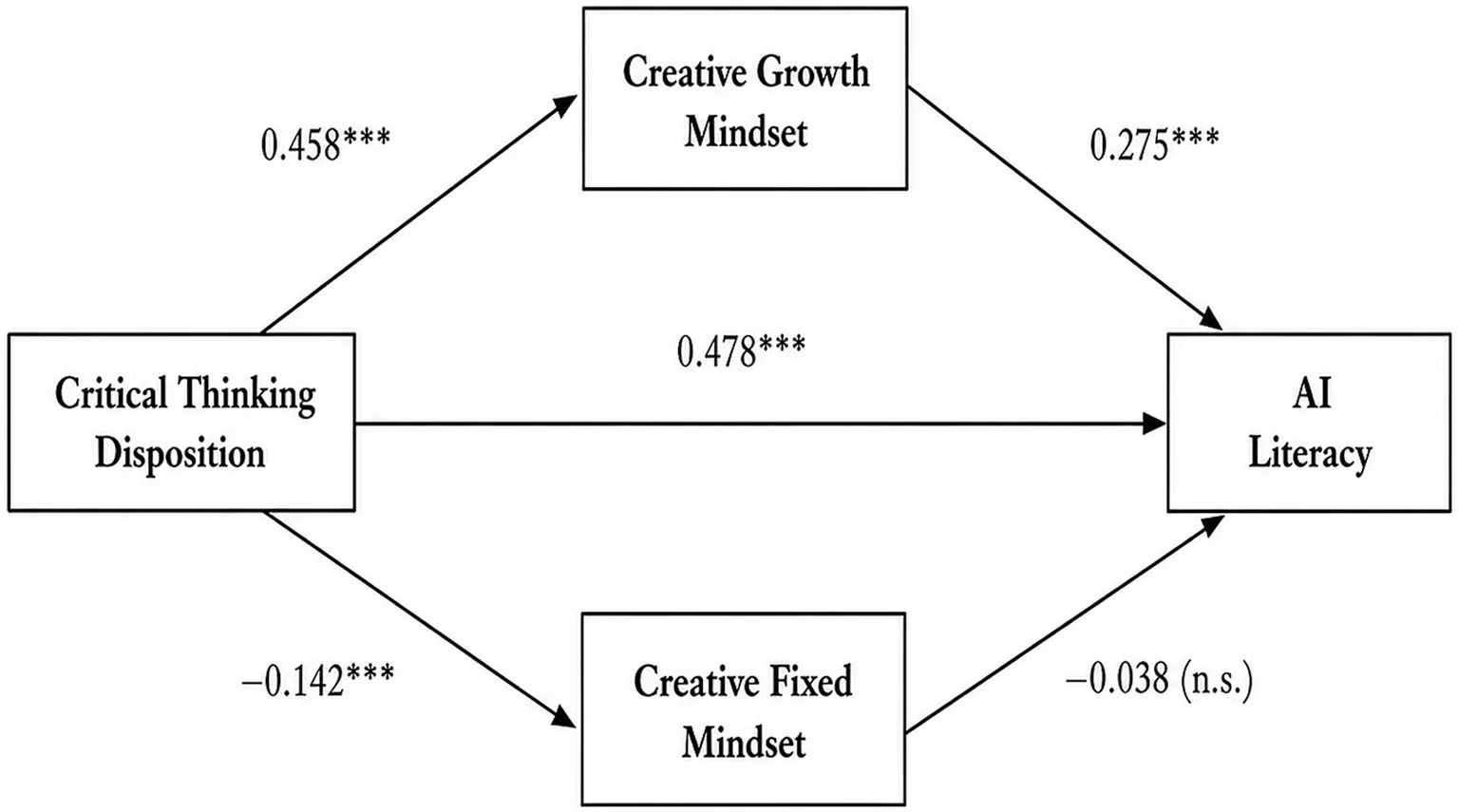
Prespecified parallel indirect-association model of creative growth mindset and creative fixed mindset between critical thinking disposition and AI literacy. ***p < 0.001.

**Table 5:** Parallel indirect associations through creative growth mindset and creative fixed mindset between critical thinking disposition and AI literacy.

Effect	Effect value	SE	95% CI	*P*	Proportion of total association (%)
Specific indirect association via creative growth mindset	0.126	0.031	0.069–0.191	<0.001	20.69
Specific indirect association via creative fixed mindset	0.005	0.006	−0.008–0.020	0.414	0.82
Total indirect association	0.131	0.031	0.076–0.191	<0.001	21.51
Direct association (c′)	0.478	0.063	0.354–0.602	<0.001	78.49
Total association (c)	0.609	0.051	0.508–0.710	<0.001	100

## 4 Discussion

This study found a mean AI literacy score of 55.71 ± 7.34 among undergraduate nursing students. Among the dimensions, the Ethics dimension scored relatively higher, while the Awareness dimension scored relatively lower. The observed AI literacy score and dimensional pattern can be considered alongside previous findings among undergraduate nursing students in Northeast China (6). A multinational study among college students also showed that students had high interest and positive attitudes toward using AI, but their AI literacy only reached a basic level (10). These findings suggest that although undergraduate nursing students have acquired a certain foundation in AI exposure and usage, they still have deficiencies in understanding AI concepts and deeper cognition. These results align with global viewpoints on AI education. The UNESCO AI Competency Framework highlights that AI literacy encompasses not just technical abilities but also critical assessment, ethical judgement, responsible application, and continuous education (3). International nursing education underscores the necessity of incorporating AI literacy into nursing programs, while fostering students’ abilities in critical assessment, ethical AI application, and the skills essential for AI-enhanced professional practice (21–24). The comparatively elevated score in the Ethics dimension can be ascribed to the enduring focus in nursing school on professional proficiency, risk mitigation, and ethical standards, leading students to exercise greater caution regarding AI usage. In contrast, the relatively lower score in the Awareness dimension indicates that nursing students still have insufficient understanding of the basic principles, application boundaries, and potential limitations of AI. Previous studies have pointed out that AI literacy is essentially a competency composed of cognition, application, evaluation, and ethics, and that college students often tend to be relatively weaker in the technical understanding or cognitive dimension, which is largely consistent with the finding of “the lowest Awareness dimension” in this study (25). This suggests that, despite being proficient in using generative AI tools, students may still have limited understanding of how these systems generate outputs and may not adequately recognize their limitations, including potential biases and inaccurate or fabricated information (26). Therefore, when conducting AI-related education, nursing institutions should not only emphasize “how to use” but also strengthen training in “understanding principles, identifying limitations, and making sound judgments,” so as to promote the coordinated development of all dimensions of AI literacy.

This study showed that interest, attitude, and AI education experience were all statistically significant in the univariate analysis. However, in the regression model, attitude and AI education experience retained stable explanatory power, while interest did not enter the regression equation. This suggests that interest may primarily reflect surface-level motivation, which facilitates nursing students’ exposure to and engagement with AI learning, but its effect still needs to be realized through more stable attitudes, educational experiences, and cognitive abilities. Relevant studies have shown that a positive attitude toward AI helps enhance users’ openness to and investment in new technologies (27). At the same time, systematic AI education can provide students with the necessary knowledge frameworks, practical opportunities, and normative guidance, thereby promoting the development of higher levels of AI literacy (6).

This study also found significant differences in AI literacy across frequencies of AI technology use. The descriptive pattern indicated generally higher AI literacy among students with more frequent AI exposure than among those reporting very limited use. However, the pattern was not strictly linear across all frequency categories. This finding is broadly consistent with recent evidence among Chinese nursing students identifying frequency of AI use as a significant factor associated with AI literacy (28). Repeated engagement with AI technologies may provide greater opportunities for students to become familiar with their functions, applications, and limitations. However, frequency of use alone should not be interpreted as equivalent to high-quality or critically informed AI engagement, as the purpose, depth, and context of AI use may also be important. Therefore, nursing education should combine opportunities for meaningful AI practice with structured guidance on critical evaluation, ethical use, and appropriate application of AI-generated information.

This study’s results indicated that the critical thinking disposition had a positive correlation with AI literacy (*r* = 0.544) and continued to be a significant predictor in the regression model. This indicates that critical thinking could be a significant cognitive prerequisite for AI literacy among nursing students. This finding is consistent with previous research highlighting the important role of critical thinking in the context of generative AI (10). Although generative AI can rapidly generate text and suggestions, its output does not equate to reliable evidence and may still contain logical flaws, amplified biases, and ethical risks (3). In nursing learning contexts, the information students encounter is closely related to professional knowledge, clinical judgment, and patient safety. Therefore, possessing the ability to use AI tools alone is insufficient; more importantly, students need to be able to discriminate, compare, verify, and prudently adopt AI-generated content. Previous studies have found that students with stronger critical thinking tend to adopt reflective, cautious, and collaborative approaches to using AI, rather than uncritically accepting AI suggestions (2). As a result, nursing students who possess a stronger critical thinking disposition are more likely to actively consider the sources of information and the quality of the evidence, see possible issues, and consequently lessen their passive acceptance of AI output, thereby improving their AI literacy. This argues that in order for students to genuinely develop the competency of “being able to use AI and, more importantly, capable of evaluating AI,” AI literacy cultivation in nursing education should be combined with evidence-based thinking training, case analysis, and ethical conversations. Additionally, a slight positive association between AI literacy and a creative fixed mindset was found. Although this finding differs from the theoretical expectation that fixed beliefs may hinder learning, the association was small and did not remain statistically significant in the multivariable regression analysis. One possible explanation is that creative fixed mindset reflects only one aspect of students’ learning beliefs and may coexist with positive attitudes toward AI or prior educational experiences. Therefore, this association should be interpreted cautiously, and future studies are needed to clarify its underlying mechanisms.

This study found a statistically significant indirect association between critical thinking disposition and AI literacy through creative growth mindset; however, the corresponding indirect association through creative fixed mindset was not statistically significant. Both creative growth mindset and creative fixed mindset were retained in the prespecified parallel model irrespective of statistical significance, thereby allowing both theoretically specified pathways to be evaluated and reported transparently. This pattern is consistent with, but does not establish, the proposed pathway based on Social Cognitive Theory and creative growth mindset research (8, 13, 15, 17). While students with better creative growth mindset beliefs may also report greater persistence, feedback seeking, and approach change, students with stronger critical thinking dispositions may be more likely to identify limitations in AI-generated content. Reverse or reciprocal interactions are still possible, though, because all variables were assessed at a single point in time. Therefore, to ascertain temporal ordering and assess the suggested mechanism, longitudinal and intervention studies are required. When interpreting these results, the sampling approach should be taken into account. Convenience sampling was used to pick participants from three Henan Province universities, which could have led to selection bias. It’s possible that students who willingly answered an online survey about artificial intelligence were more interested in, familiar with, or had access to digital technologies than those who did not. Furthermore, differences across regions and institutions—including AI infrastructure, curriculum design, faculty expertise, educational resources, and access to generative AI tools—may influence students’ AI literacy. Consequently, the present findings may not represent undergraduate nursing students from other provinces, rural settings, or institutions with different educational characteristics. In addition, all participants were third-year nursing students; therefore, the findings may not necessarily generalize to first-, second-, or final-year students, whose exposure to clinical training, AI-related education, and technology-supported learning may differ by stage of study. Future studies should use stratified or probability-based sampling across multiple geographical regions, different types of nursing institutions, and multiple academic years to improve representativeness and external validity and to examine whether AI literacy and its associated factors vary across stages of nursing education.

## 5 Conclusion

The findings indicate that perceived AI literacy was significantly associated with attitude toward AI, prior AI education, critical thinking disposition, and creative growth mindset. The cross-sectional indirect-association analysis further showed that creative growth mindset statistically accounted for part of the association between critical thinking disposition and perceived AI literacy. These findings should be interpreted as concurrent statistical associations rather than evidence that one variable causes change in another. These findings provide useful insights for shifting AI education in higher education from a technology-oriented approach to a “cognition–ethics–mindset synergistic” approach. However, this study has certain limitations. Furthermore, AI literacy was assessed using self-report instruments that reflect students’ perceived AI literacy rather than objectively measured AI competence. Therefore, the findings should be interpreted as participants’ self-perceptions and may not fully represent their actual ability to apply AI knowledge and skills in authentic educational or clinical settings. Temporal ordering and causal linkages cannot be established because this study used a cross-sectional design and examined every variable at once. The observed indirect effect should not be taken as proof of a causal mediating mechanism since reverse or reciprocal correlations cannot be ruled out. To test the temporal sequence of the suggested correlations, longitudinal and intervention studies are necessary. However, this study still recommends that when it comes to AI education, nursing schools should move away from simple tool training and toward the synergistic development of conceptual understanding, critical evaluation, ethical judgement, and growth beliefs. To further investigate the developmental mechanisms of AI literacy among nursing students and the effects of educational interventions, future research could perform longitudinal or intervention studies in multi-regional and multi-level samples, incorporating data from contextualised tasks and behavioural evaluations. This would provide a foundation for AI education in nursing talent cultivation.

## Data Availability

The original contributions presented in the study are included in the article/supplementary material, further inquiries can be directed to the corresponding author/s.
